# The C5a-C5aR1 axis facilitates blood-brain barrier disruption induced by EV-A71 infection through neutrophil activation

**DOI:** 10.1371/journal.ppat.1014487

**Published:** 2026-08-05

**Authors:** Peiyu Zhu, Xiaofeng Zeng, Xin Yuan, Zijie Li, Wangquan Ji, Dong Li, Tiantian Sun, Haiyan Yang, Shuaiyin Chen, Jinzhao Long, Fang Wang, Yaodong Zhang, Adong Shen, Weiguo Zhang, Yuefei Jin, Guangcai Duan

**Affiliations:** 1 Department of Epidemiology, College of Public Health, Zhengzhou University, Zhengzhou, China; 2 Henan International Joint Laboratory of Children’s Infectious Diseases, Children’s Hospital Affiliated to Zhengzhou University, Henan Children’s Hospital, Zhengzhou Children’s Hospital, Zhengzhou, China; 3 Department of Forensic Medicine, School of Forensic Medicine, Kunming Medical University, Kunming, China; 4 Department of Infection Control, Henan Provincial People’s Hospital, People’s Hospital of Zhengzhou University, Zhengzhou, China; 5 Key Laboratory of Major Diseases in Children, Ministry of Education, National Center for Children’s Health, Beijing Children’s Hospital, Beijing, China; 6 National Key Laboratory of Immunity and Inflammation, Suzhou Institute of Systems Medicine, Chinese Academy of Medical Sciences & Peking Union Medical College, Suzhou, China; 7 Pingyuan Laboratory, Xinxiang, China; Oklahoma State University College of Osteopathic Medicine, UNITED STATES OF AMERICA

## Abstract

Enterovirus A71 (EV-A71), a member of the genus *Enterovirus* within the family *Picornaviridae*, induces neuroinflammation; however, the underlying mechanisms remain incompletely understood. This study demonstrates that the C5a-C5aR1 axis plays a pivotal role in EV-A71-induced blood-brain barrier (BBB) disruption and neuroinflammation, primarily by regulating neutrophil migration. Using human brain specimens and a mouse model, we observed pronounced inflammatory cell infiltration in the brainstem and BBB disruption following EV-A71 infection. Immunofluorescence analysis revealed robust activation of the C5a-C5aR1 axis in fatal EV-A71 cases. Notably, C5aR1 knockout (KO) mice displayed reduced Evans blue extravasation and preserved tight junction protein expression after infection. Immunopathological examination of fatal human cases further confirmed perivascular neutrophil (CD177⁺) infiltration in the brainstem. Importantly, C5aR1 deficiency significantly attenuated neutrophil accumulation and neutrophil extracellular trap (NET) release. Given that peptidylarginine deiminase 4 (PAD4) is a key enzyme driving NET formation, we generated neutrophil-specific PAD4 knockout mice (PAD4 Ne-KO) by crossing S100A8-Cre and PAD4^fl/fl^ lines. As anticipated, neutrophil-specific PAD4 deletion or pharmacological NET blockade substantially ameliorated BBB injury and neuroinflammation following EV-A71 infection. Overall, our findings underscore a critical role for the C5a-C5aR1-neutrophil/NETs pathway in EV-A71 encephalitis pathogenesis and support its targeting as a therapeutic strategy for critically ill patients.

## Author summary

Enterovirus A71 (EV-A71) is a member of the *Picornaviridae* family, genus *Enterovirus*, and species *Enterovirus A.* It is characterized by pronounced neurotropism, resulting in severe neurological complications-including meningitis and brainstem encephalitis that pose a significant threat to pediatric health worldwide. However, the pathogenic mechanisms underlying EV-A71 neurovirulence remain unclear. Here, we use human brain specimens to demonstrate that neutrophil infiltration in the brainstem is accompanied by pronounced activation of the C5a-C5aR1 axis. Using C5aR1 knockout mice, we establish that the C5a-C5aR1 axis is essential for blood-brain barrier (BBB) disruption and neuroinflammation induced by EV-A71, primarily by governing neutrophil trafficking. Furthermore, by employing PAD4-deficient mice (PAD4 Ne-KO) and the NET inhibitor sivelestat, we confirm that neutrophil-intrinsic PAD4 loss or pharmacological NET blockade substantially attenuates BBB injury and neuroinflammation following EV-A71 infection. Our study highlights a critical role for the C5a-C5aR1-neutrophil/NETs pathway in EV-A71 encephalitis pathogenesis and provides a mechanistic rationale for targeting this pathway as a therapeutic strategy for critically ill patients with HFMD.

## Introduction

Enterovirus A71 (EV-A71), belonging to the genus *Enterovirus* within the family *Picornaviridae*, is a major etiological agent of severe neurological disorders in children [1]. Over the past three decades, EV-A71 has has triggered recurrent outbreaks across the Western Pacific, claiming thousands of young lives [2–4]. Although an inactivated EV-A71 vaccine was licensed in China in 2016, ongoing viral evolution continues to threaten pediatric health [5]. The virus spreads primarily via the fecal-oral route, respiratory droplets, and contact with contaminated secretions, disproportionately affecting children under five years of age [1,6]. Upon entry, EV-A71 initially replicates in the small intestine and lymph nodes before disseminating systemically [1,7]. In some cases, EV-A71 may invade the central nervous system (CNS), leading to severe complications such as neurogenic pulmonary edema secondary to brainstem encephalitis [8]. Autopsy results showed EV-A71 antigen in the brainstem, diencephalon, cerebellar dentate nucleus, and anterior horn cells of the spinal cord. Notably, viral antigens were absent in some damaged nerve cells and undetectable in areas with severe inflammation [9]. These findings suggest that the robust inflammatory response triggered by viral invasion, rather than direct cytopathic damage, plays a central role in driving the pathogenesis of EV-A71 encephalitis [10].

The blood-brain barrier (BBB) represents a critical anatomical structure within the brain. BBB dysfunction has been implicated in a broad spectrum of neurological disorders, ranging from acute conditions such as viral encephalitis to chronic disorders like Alzheimer’s disease [11]. Notably, several flaviviruses-including Zika virus (ZIKV) [12], West Nile virus (WNV) [13], and Japanese encephalitis virus (JEV) [14]-induce BBB dysfunction by compromising barrier integrity and downregulating tight junction proteins (TJs) expression. A recent study has shown that microvesicles facilitate the transport of EV-A71 virions across the BBB, ultimately leading to brain injury in mice [15]. In *vitro* BBB models further demonstrate that extracellular vesicles carrying EV-A71 can modulate the expression of TJs [16]. Moreover, EV-A71-induced miR-3473a has shown to regulate focal adhesion and leukocytes transendothelial migration, implicating it in virus-mediated BBB disruption [17]. Collectively, these lines of evidence underscore that the disruption of the BBB caused by EV-A71 invasion plays a crucial role in the pathogenesis of encephalitis.

The complement system, a cascade of interacting plasma and membrane-bound proteins, is integral to both innate and adaptive immunity [18]. Although the liver is the primary source of circulating complement components, resident neural cells-including microglia and astrocytes-can locally synthesize complement factors, regulators, and receptors, establishing functional complement pathways within CNS [19]. This system serves as a key local surveillance mechanism in the brain, protecting neural tissue and maintaining homeostasis and repair [20]. Proteolytic cleavage products generated during complement activation regulate immune cell extravasation and migration to inflammatory sites [21,22]. Notably, our previous work has demonstrated that the complement fragment C5a and its receptor C5aR1 are key drivers of neuroinflammation induced by EV-A71, with EV-A71 activating astrocytes via the C5a-C5aR1-p38 MAPK pathway [23]. Accumulating evidence further implicates complement activation in BBB disruption and subsequent brain pathology [24,25]. In the present study, we investigated the impact of EV-A71 infection on BBB integrity using human specimens and a murine infection model, with a focus on the C5a-C5aR1 axis. Our findings reveal that the C5a-C5aR1 axis drives BBB disruption during EV-A71 infection by recruiting and activating neutrophils. Blocking neutrophil extracellular trap (NET) release significantly attenuates EV-A71-induced BBB permeability. These results elucidate the neuroimmune mechanisms underlying EV-A71 encephalitis and provide a rationale for targeting the C5a-C5aR1 axis in critically ill patients.

## Results

### Pronounced perivascular neutrophil infiltration is a hallmark pathological feature of EV-A71 encephalitis

**Fig 1:**
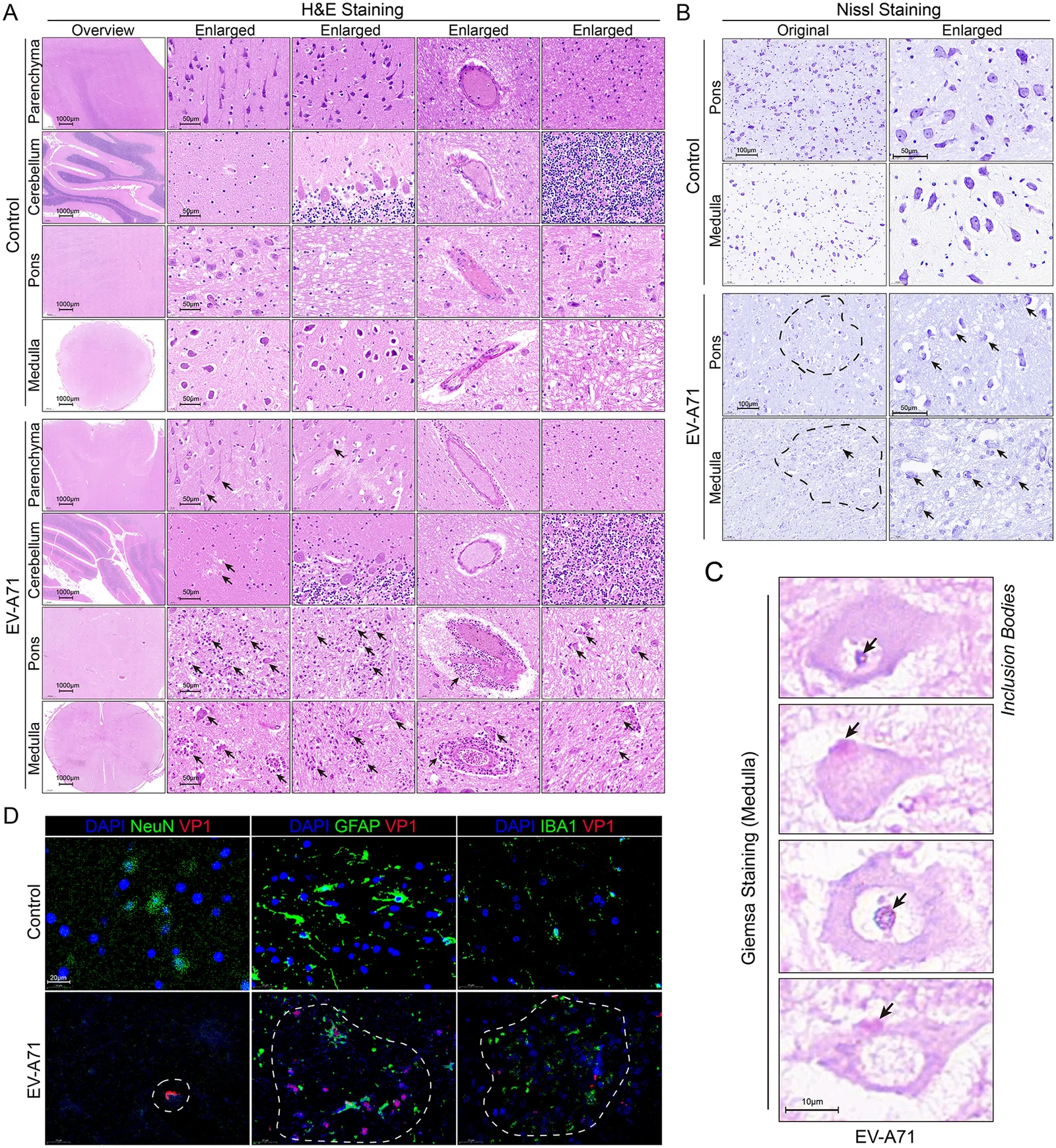
Immunopathological characteristics of brain tissues in fatal EV-A71 cases. Six cases of childhood fatalities were obtained from the forensic identification center, Kunming Medical University. Among these, three cases were attributed to EV-A71 infection, while the remaining three cases resulted from other non-infectious diseases (without concurrent encephalitis). **(A-D)**: Histological evaluations of brain specimens were conducted using H&E staining, Nissl staining, Giemsa staining, immunofluorescence. Scale bars: 50 μm **(A)**, 100 μm **(B)**, 10 μm **(C)**, and 20 μm **(D)**. The arrows and dotted lines denote neuronal damage **(A, B)**, infiltration of inflammatory cells **(A)**, proliferation of glial cells **(A)**, the presence of viral inclusions **(C)**, and viral antigens **(D)**.

**Fig 2:**
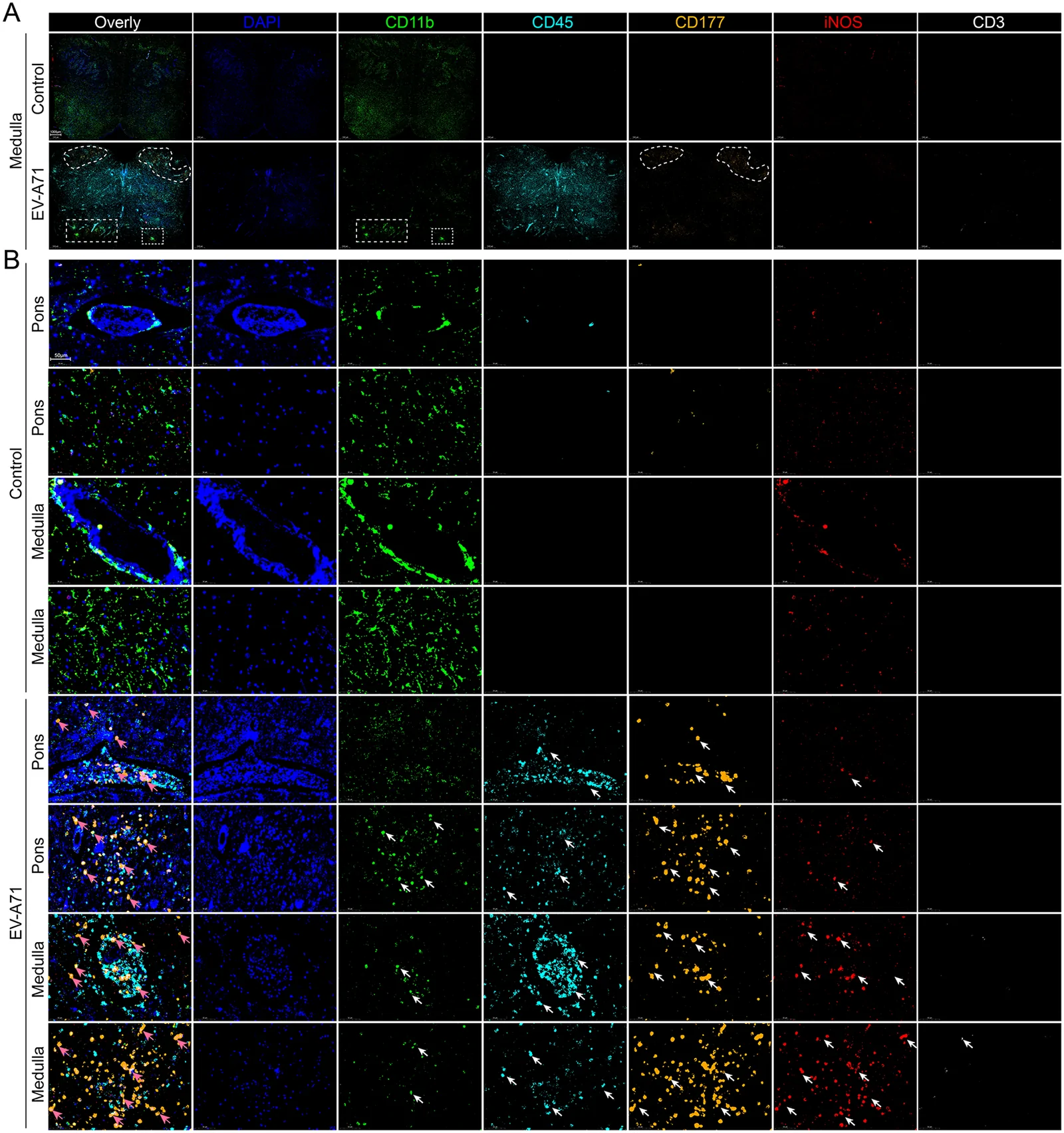
Immune cell profiling in brain specimens from fatal EV-A71 cases. Brain tissues of fatal EV-A71 cases were analyzed by multiple immunofluorescence staining. **(A)**: Overview of the brainstem. Bar = 1000 μm. **(B)**: A localized field of vision within the brainstem. Bar = 50 μm. The white arrows and the white dotted areas highlighted in white indicate the infiltration of leukocytes, and the red arrow indicates the co-localization of the fluorescent signal with leukocytes.

To characterize EV-A71-induced brain pathology, we examined postmortem brain specimens from fatal EV-A71 encephalitis cases. As illustrated by Fig 1A and 1B, EV-A71 infection caused predominant brainstem damage, characterized by perivascular cuffing, glial nodule formation, and neuronophagia. Viral inclusion bodies were observed in neurons (Fig 1C), and viral antigens were identified in nerve cells (Fig 1D). Given the prominent inflammatory infiltration, we profiled immune cell phenotypes in brain tissues from fatal cases by immunofluorescence, using CD45 (pan-leukocyte), CD11b (monocytes/NK cells/microglia), CD117, iNOS (macrophages/MDSCs), and CD3 (T cells) as markers. Compared with controls, fatal EV-A71 cases exhibited substantial inflammatory cell infiltration in the anterior and posterior funiculi of the medulla oblongata (Fig 2A), with pronounced accumulation of CD11b^+^, CD117^+^, and CD45^+^ cells. Further examination of the brainstem parenchyma and perivascular regions revealed a significant influx of CD45^+^CD117^+^ neutrophils from the bloodstream, resulting in perivascular infiltration (Figs 2B, **S1**). Notably, a subset of CD177^+^ neutrophils co-localized with iNOS (Fig 2), consistent with iNOS incorporation into NETs. These findings demonstrate that pronounced perivascular neutrophil infiltration is a hallmark pathological feature of EV-A71 encephalitis.

### EV-A71 infection compromises BBB integrity in humans and animals

**Fig 3:**
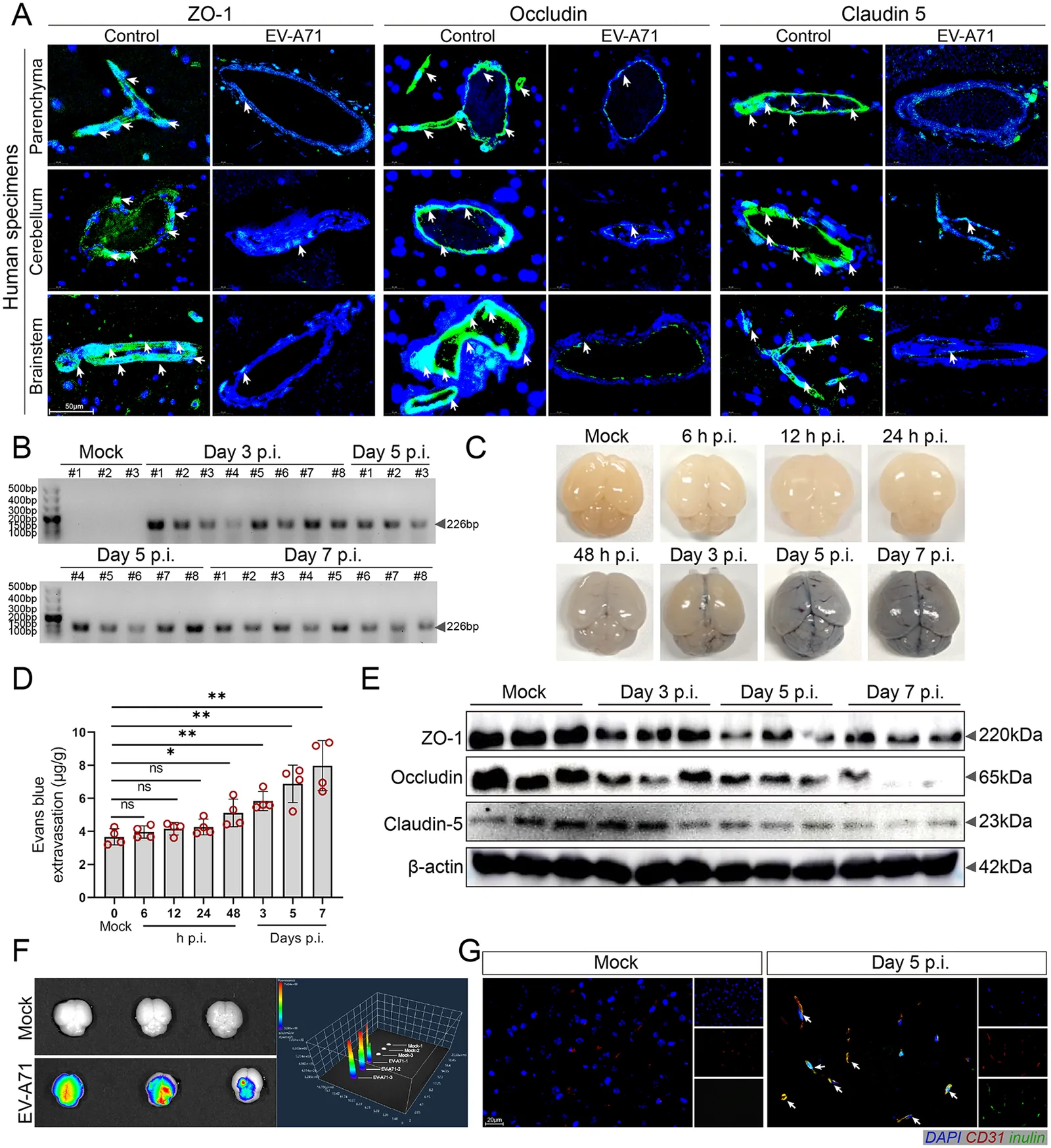
EV-A71 compromises BBB integrity in humans and animal models. **(A)**: The expression of TJs (ZO-1, Occludin, and Claudin-5) in the vessels of human specimens (n = 3 for each group) was assessed using immunofluorescence. Bar = 50 μm. Blue represents the cell nucleus and green represents ZO-1, Occludin, or Claudin-5. White arrows indicate protein-positive signals. **(B)**: EV-A71 VP1 expression was detected by RT-qPCR. **(C-D)**: The Evans blue extravasation assay was employed to assess the permeability of the BBB. The images depicting Evans blue extravasation and its concentration (n = 4). **(E)**: The expression levels of TJs in brain tissues were evaluated by Western blotting (n = 3). **(F)**: EV-A71-infected mice (n = 3) received FITC-inulin injections at Day 5 p.i.. Brains were imaged *ex vivo* 2 h post-injection. **(G)**: FITC-inulin co-localization with CD31 was assessed by immunofluorescence. Blue represents the cell nucleus, and green represents inulin, and red represents CD31. Bar = 50 μm. White arrows indicate co-localization sites. *P < 0.05; **P < 0.01, EV-A71-infected mice vs mock-infected mice.

Neutrophil infiltration in the brain is a key driver of BBB disruption and encephalitis. To characterize EV-A71-induced BBB damage, we examined brain specimens from fatal human cases and EV-A71-infected mice. Compared with controls, fatal EV-A71 cases significant exhibited marked degradation of vascular junction proteins (ZO-1, Occludin, and Claudin-5) in the parenchyma, cerebellum, and brainstem (Figs 3A and S2). We next assessed BBB permeability in EV-A71-infected mice. As shown in Fig 3, viral replication was detected in the brains of infected mice (Fig 3B), and Evans blue extravasation in brain tissue progressively increased with prolonged infection (Fig 3C and 3D). Western blot analysis further revealed a time-dependent decrease in ZO-1, Occludin, and Claudin-5 levels in mouse brains (Fig 3E). Intravenous administration of FITC-labeled inulin enabled direct visualization of BBB breakdown, revealing markedly elevated fluorescence intensity in the brainstem of infected mice (Fig 3F). Subsequent immunofluorescence staining confirmed perivascular distribution of inulin (Fig 3G). Taken together, these findings demonstrate that EV-A71 infection compromises BBB integrity in humans and mice.

### The C5a-C5aR1 axis drives BBB disruption in EV-A71 Infection

**Fig 4:**
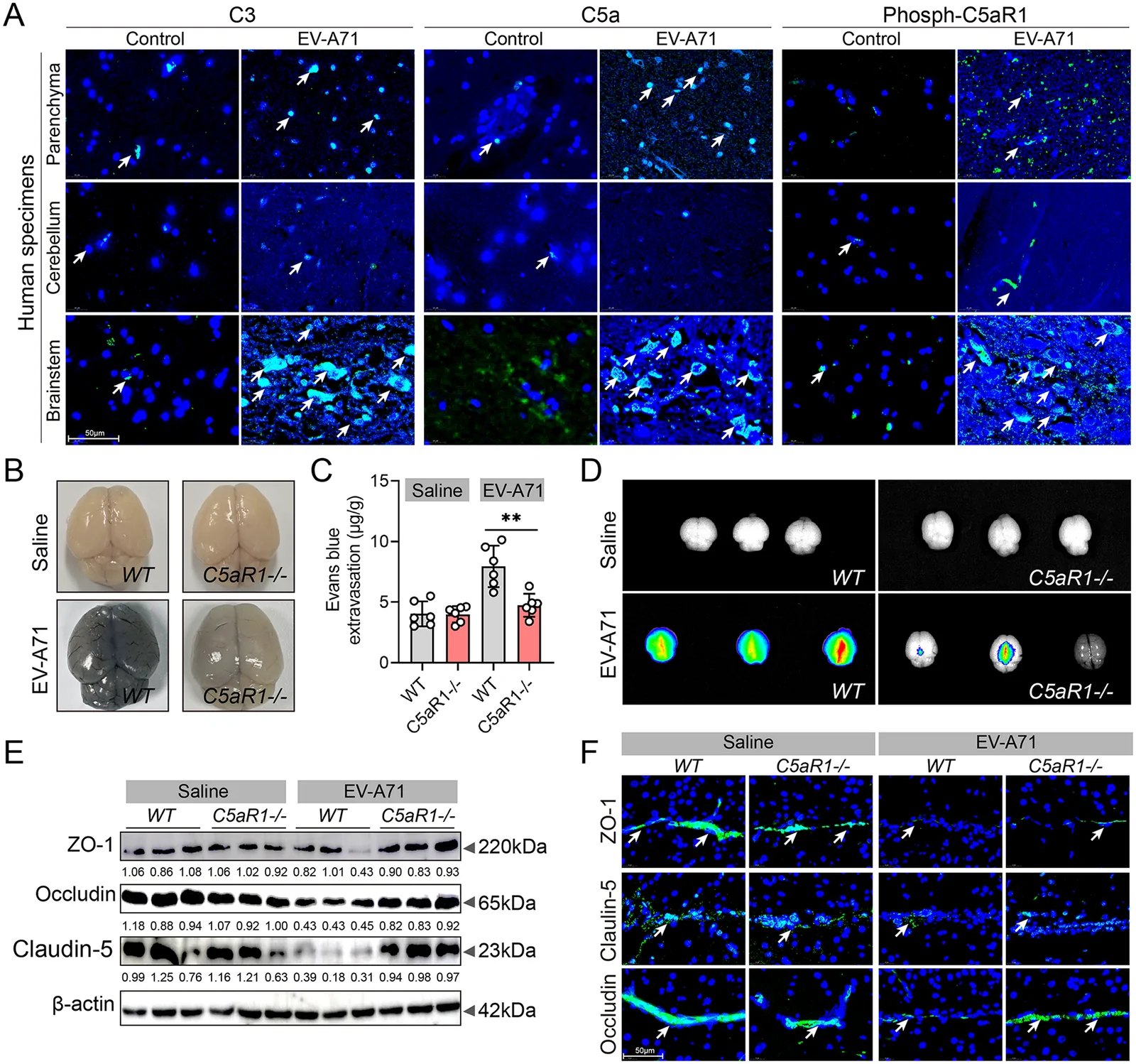
The importance of the C5a-C5aR1 axis in BBB disruption in EV-A71 infection. **(A)**: Complement protein expression (C3, C5a, and phosph-C5aR1) in brain tissues from fatal EV-A71 cases and fatal controls (n = 3 for each group) were assessed using immunofluorescence. Bar = 50 μm. Blue, nuclei; green, C3, C5a, or phospho-C5aR1. White arrows indicate positive signals. Five-day-old WT and C5aR1 KO mice were i.p. injected with 2.86 × 10⁶ TCID₅₀ of EV-A71 or saline, and euthanized at Day 7 p.i.. **(B-C)**: Evans blue extravasation in mouse brains (n = 6) at Day 7 p.i.. Representative images and quantification. **(D)**: EV-A71-infected WT and C5aR1 KO mice (n = 3) received FITC-inulin injections at Day 5 p.i.. Brains were imaged *ex vivo* 2 h post-injection. **(E-F)**: Tight junction protein levels in brain tissues of EV-A71-infected WT and C5aR1 KO mice (n = 3) were evaluated by Western blotting and immunofluorescence. Bar = 50 μm. Blue, nuclei; green, ZO-1, Occludin, or Claudin-5. White arrows indicate co-localization sites or positive signals. **P < 0.01 vs. WT mice.

Our findings demonstrate that plasma levels of C3 and C5a are significantly elevated in children with EV-A71 infection [23], accompanied by complement deposition in the astrocytes of EV-A71-infected mice brains (S3 Fig). To further evaluate complement activation in the brain, we performed immunofluorescence staining assess C3, C5a, and phospho-C5aR1 expression in the brainstem, cerebellum, and parenchyma of fatal EV-A71 cases (Figs 4A and S4). As expected, complement levels were significantly elevated in the brains of fatal EV-A71 cases, particularly within the brainstem. To investigate the role of the C5a-C5aR1 axis in BBB disruption, we infected C5aR1 KO mice. C5aR1 deficiency significantly attenuated Evans blue extravasation in the brains of mice after infection (Fig 4B and 4C), with concordantly reduced inulin permeability (Fig 4D). Western blotting and immunofluorescence staining further demonstrated that C5aR1 knockout attenuated EV-A71-induced reductions in ZO-1, Occludin, and Claudin-5 expression (Fig 4E and 4F). Collectively, our findings establish that the C5a-C5aR1 axis plays an important role in BBB disruption during EV-A71 infection.

### The C5a-C5aR1 axis drives neutrophil recruitment to EV-A71-infected brains

**Fig 5:**
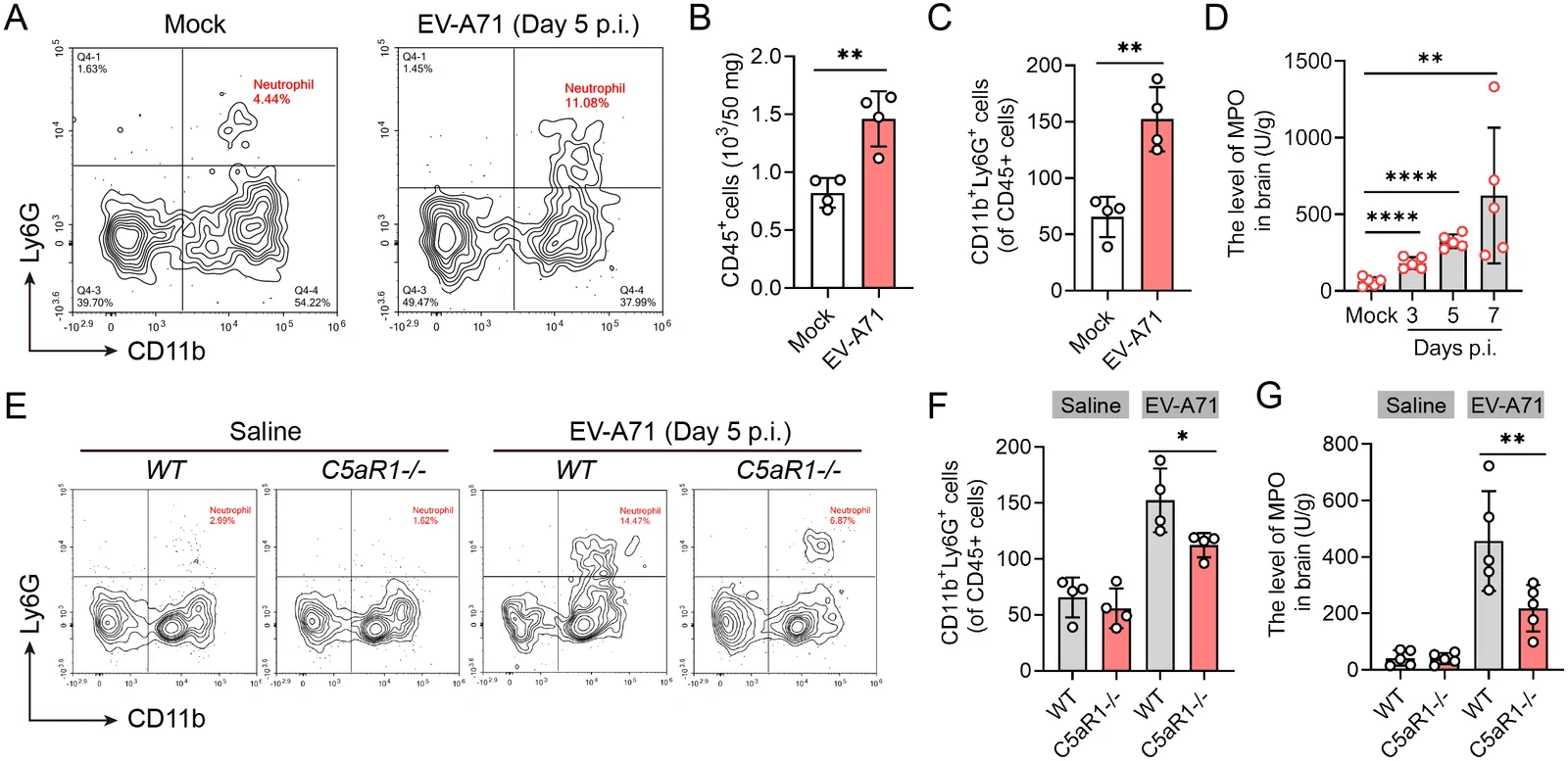
The C5a-C5aR1 axis drives neutrophil recruitment to EV-A71-infected brains. Five-day-old WT (C57BL/6J) mice were i.p. injected with 2.86 × 10^6^ TCID_50_ EV-A71 or saline and euthanized at Day 5 p.i.. **(A)**: Representative FACS plots of brain-infiltrating neutrophils. Leukocytes (CD45^+^, **B**) and neutrophils (CD11b^+^Ly6G^+^, **C**) in brain tissues were quantified by FACS (n = 4). **(D)**: Brain MPO levels at Day 3, 5, and 7 p.i. were measured using a commercial kit (n = 5). Five-day-old WT and C5aR1 KO mice were i.p. injected with 2.86 × 10^6^ TCID_50_ EV-A71 or saline, and sacrificed at Day 5 p.i.. **(E)**: Representative FACS plots of brain-infiltrating neutrophils. Neutrophil counts (**F**) were detected by FACS (n = 4), and MPO levels (**G**) were measured using a commercial kit at Day 5 p.i. (n = 5). *P < 0.05; **P < 0.01, ****P < 0.0001 vs. saline controls.

During viral infections, the C5a-C5aR1 axis plays a crucial role in mediating the migration of immune cells, particularly neutrophil recruitment, thereby exacerbating tissue damage. As shown in Fig 5A, both CD45^+^ cells and CD11b^+^Ly6G^+^ neutrophils were markedly increased in the brains of EV-A71-infected WT mice (Fig 5B and 5C). Consistently, MPO, neutrophil elastase (NE)-DNA, CitH3 and dsDNA were elevated in EV-A71-infected brains (Figs 5D and **S5**), with MPO levels increasing in a time-dependent manner. We previously demonstrated that C5aR1 KO mice exhibited a lower viral load in the brain and blood, along with an attenuated inflammatory response, during EV-A71 infection (**S6 Fig**) [23]. To determine whether the C5a-C5aR1 axis governs neutrophil infiltration, we performed additional experiments using C5aR1 KO mice. As expected, both neutrophil numbers (Fig 5E and 5F) and MPO concentrations (Fig 5G) in C5aR1 KO mice were significantly attenuated in C5aR1-deficient mice. Overall, these findings indicate that the activation of the C5a-C5aR1 axis drives neutrophil recruitment and activation in the brain following EV-A71 infection.

### Genetic NETs ablation attenuates EV-A71-induced neuropathology and BBB hyperpermeability

**Fig 6:**
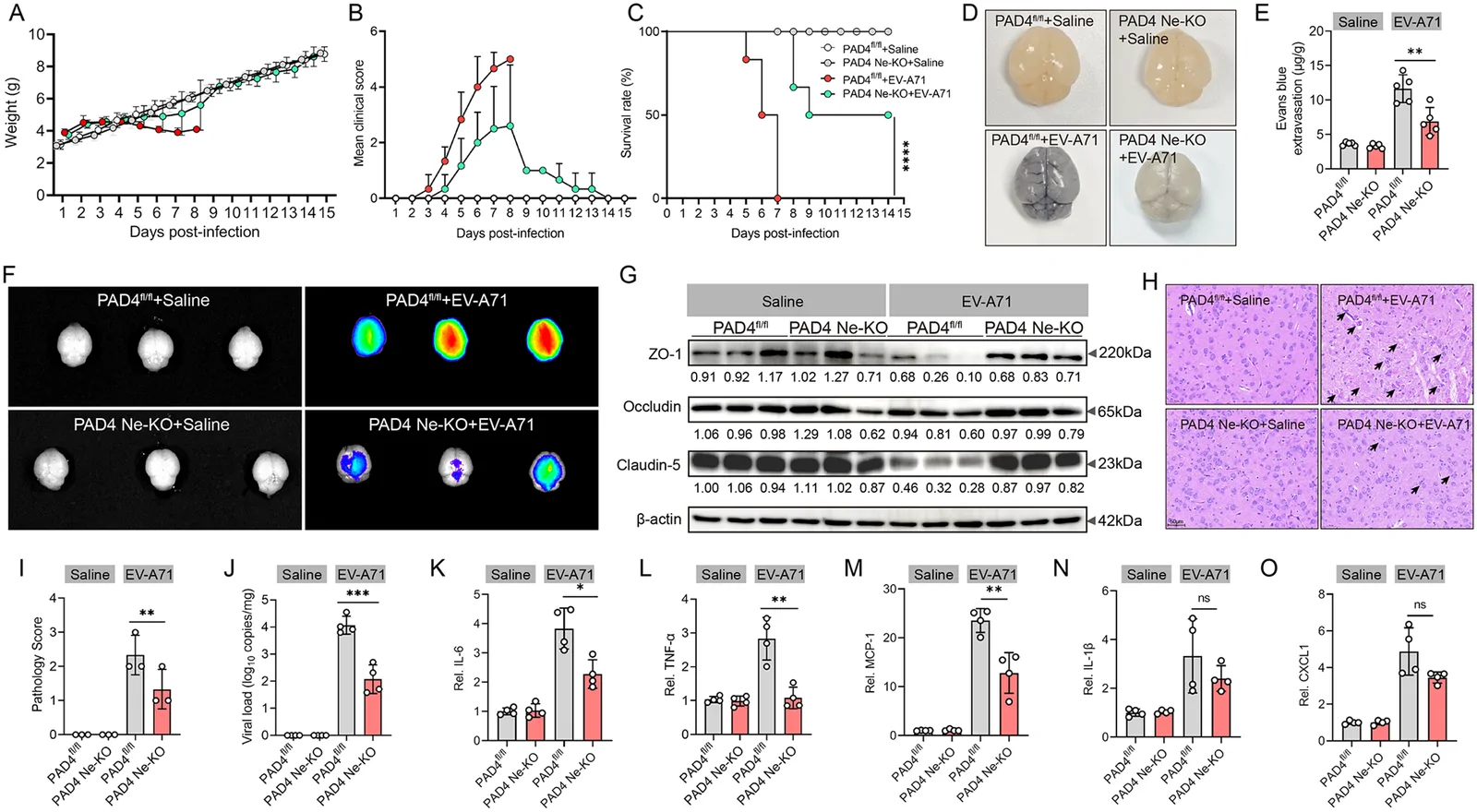
Genetic NETs ablation reduces EV-A71-induced neuropathology and BBB hyperpermeability. Five-day-old PAD4 Ne-KO and PAD4^fl/fl^ mice were i.p. inoculated with 2.86 × 10^6^ TCID_50_ EV-A71 or saline, and sacrificed at Day 7 p.i. (n = 6). **(A)**: The body weight. **(B)**: Clinical scores. **(C)**: Kaplan-Meier survival curve. **(D)**: Representative brain images (Evans blue). **(E)**: Brain Evans blue levels (n = 5). **(F)**: Representative fluorescence images of FITC-inulin leakage. **(G)**: ZO-1, Occludin, and Claudin-5 expression in brain tissues (n = 3). (**H-I**): Representative H&E-stained brain sections (n = 3). Bar = 50 μm. Black arrows indicate neuronal damage and degeneration. **(J)**: Brain viral loads. **(K-O)**: Brain mRNA levels of IL-6, TNF-α, MCP-1, IL-1β, and CXCL1 (n = 4). *P < 0.05; **P < 0.01; ***P < 0.001; ****P < 0.0001 vs. PAD4^fl/fl^ mice.

Given that NET release in the brain (Fig 5D and 5G) followed increased neutrophil recruitment during EV-A71-induced neuroinflammation, we next investigated whether targeting NETs alone could mitigate BBB damage caused by EV-A71. To this end, we generated neutrophil-specific PAD4 knockout mice (PAD4 Ne-KO) by crossing S100A8-Cre mice with PAD4^fl/fl^ mice. Neutrophil-specific PAD4 deficiency markedly ameliorated EV-A71-induced neurological disorders, as evidenced by improved body weight, alleviated clinical signs, increased survival rate, and reduced viral load in the blood (Figs 6A-6C and **S6**). PAD4 deletion in neutrophils significantly reduced EV-A71-induced BBB hyperpermeability (Fig 6D and 6E), and attenuated FITC-inulin leakage in the brainstem (Fig 6F). Moreover, EV-A71-triggered TJs degradation was substantially mitigated (Fig 6G). We further assessed the role of NETs in modulating proinflammatory cytokine production following EV-A71 infection. PAD4 Ne-KO mice exhibited decreased neuropathogenesis (Fig 6H and 6I), reduced viral loads (Fig 6J), lower MPO concentrations (**S7 Fig**), and diminished proinflammatory cytokines (IL-6, TNF-α, MCP-1, IL-1β, and CXCL1) (Fig 6K-6O) compared with PAD4^fl/fl^ controls. Collectively, these findings indicate that NET formation contributes to EV-A71-induced neuropathogenesis and BBB disruption.

### Pharmacological NET inhibition protects the BBB against EV-A71-induced damage

**Fig 7:**
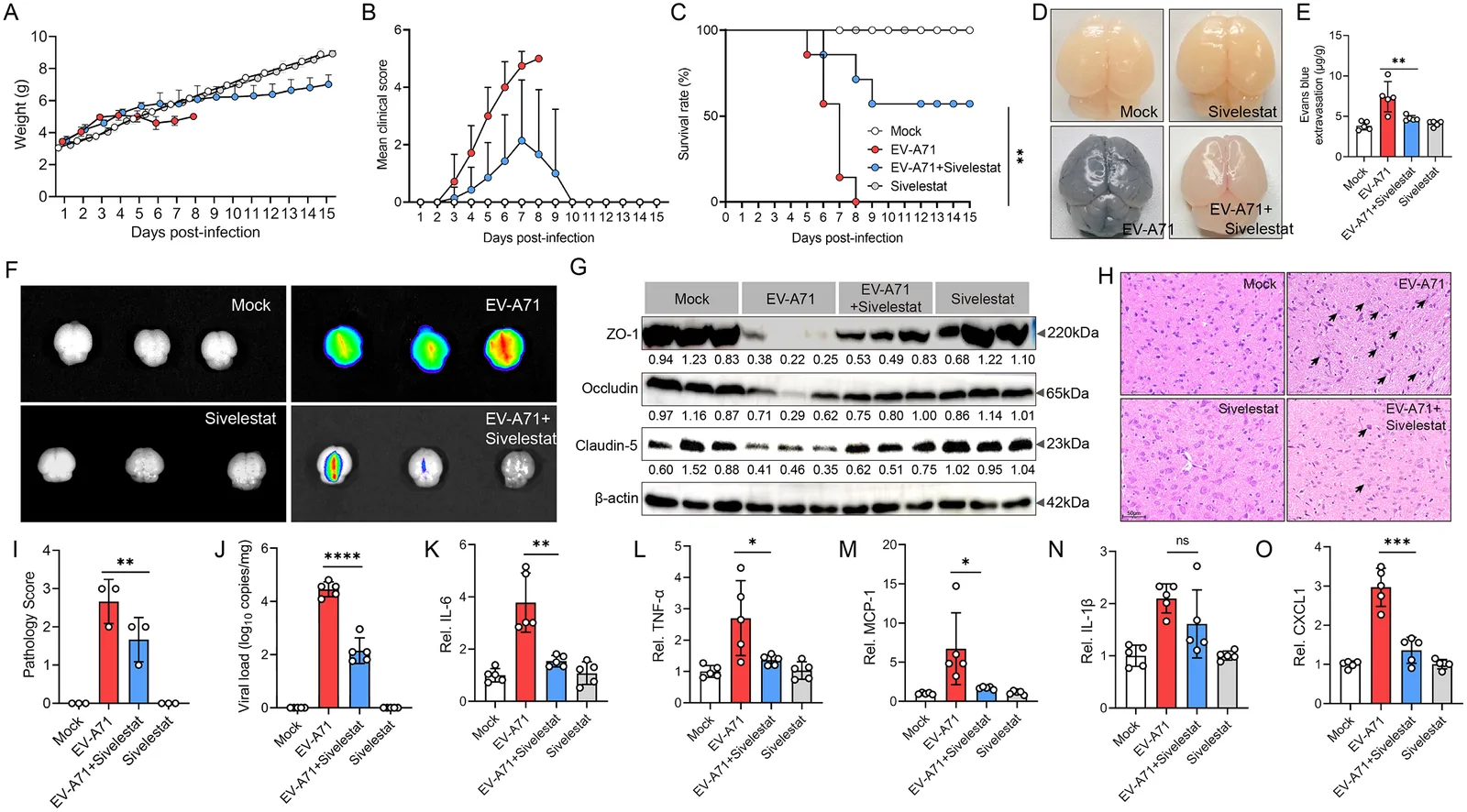
NET inhibition protects against EV-A71-induced BBB damage. Five-day-old C57BL/6J mice infected with EV-A71 received sivelestat (10.0 mg/kg b.w.) on a scheduled regimen and were euthanized at Day 7 p.i.. **(A-C)**: Body weight, clinical scores and survivals of mice were recorded from Day 1 to 15 p.i. (n = 6 ~ 7). **(D)**: Representative brain images (Evans blue). **(E)**: Brain Evans blue levels (n = 5). **(F)**: Representative fluorescence images of FITC-inulin leakage. **(G)**: ZO-1, Occludin, and Claudin-5 expression in brain tissues were analyzed by Western blotting (n = 3). (**H**, **I)**: Representative H&E-stained brain sections (n = 3). Bar = 50 μm. Black arrows indicate neuronal damage and degeneration. **(J)**: Brain viral loads. **(K-O)**: Brain mRNA levels of IL-6, TNF-α, MCP-1, IL-1β, and CXCL1 (n = 5). *P < 0.05; **P < 0.01; ***P < 0.001; ****P < 0.0001 vs. vehicle-treated mice.

Next, we evaluated the therapeutic potential of targeting neutrophil activation by administering the neutrophil elastase inhibitor sivelestat to EV-A71-infected mice. As anticipated, MPO concentrations were markedly reduced in the brains of sivelestat-treated mice (**S7 Fig**). As illustrated in Figs 7A-7C and **S6**, sivelestat mitigated clinical manifestations and blood viral load in EV-A71-infected mice, and significantly improved the survival rate and body weight. Sivelestat also attenuated Evans blue extravasation (Fig 7D and 7E) and FITC-inulin in the brain (Fig 7F), while preserving TJs integrity compromised by EV-A71 infection (Fig 7G). Furthermore, sivelestat effectively suppressed EV-A71-induced neuropathology and inflammatory responses (Fig 7H-7O**)**. Together, these findings suggest that pharmacological NET inhibition represents a promising therapeutic strategy for EV-A71 encephalitis.

## Discussion

Neurological complications caused by EV-A71 continue to be reported worldwide [26,27]. We previously demonstrated that the C5a-C5aR1 axis is a key driver of neuroinflammation during EV-A71 infection [23]. In this study, we first confirmed BBB disruption in both humans and mice following EV-A71 infection. We then leveraged human specimens, C5aR1 KO, PAD4^fl/fl^, and neutrophil-specific PAD4 knockout mice (PAD4 Ne-KO) mice to dissect the role of the C5a-C5aR1 axis in EV-A71-induced BBB damage. Our findings establish, for the first time, that the C5a-C5aR1 axis drives BBB disruption and subsequent neuroinflammation in EV-A71 infection by orchestrating neutrophil recruitment and activation. There results provide new mechanistic insights and identify potential therapeutic targets for severe HFMD.

Autopsy reports indicate that EV-A71 predominantly affects the pons and medulla (brainstem), exhibiting a spectrum of pathological changes including inflammatory cell infiltration, perivascular cuffing, glial nodules, neuronal degeneration, and necrosis [28,29]. In this study, we also observed EV-A71-induced brainstem inflammation, with inclusion bodies and viral antigens detected in a subset of neurons. Previous animal studies have demonstrated that EV-A71 is capable of direct infection of neural cells, particularly astrocytes [23,30]. The primary proposed neuroinvasive pathways for EV-A71 include retrograde axonal transport, direct transcytosis across the BBB, and a “Trojan horse” mechanism mediated by infected immune cells [1]. Among these, retrograde axonal transport is the best-characterized route by which EV-A71 gains access to the CNS. Concurrently, BBB integrity serves as a critical physiological barrier that restricts the entry of pathogens-including viruses-and other exogenous substances into the CNS [31]. Emerging evidence suggests that these two mechanisms are not mutually exclusive but rather functionally interdependent: first, EV-A71 infection, potentially initiated via retrograde axonal transport, may induce BBB dysfunction, increasing paracellular permeability and facilitating enhanced viral dissemination into the CNS; second, BBB disruption may generate a permissive neurovascular microenvironment that potentiates both the efficiency and spatial extent of retrograde axonal transport [15,32]. Collectively, these findings indicate that retrograde axonal transport and BBB compromise act synergistically to promote EV-A71 neuroinvasion. Indeed, we demonstrated BBB disruption following EV-A71 infection in both human specimens and mouse models. Similar to the JEV, which compromise the integrity of the BBB by disrupting TJs (Claudin-5, Occludin, and ZO-1) [14,33], EV-A71 has also been shown to induce early Evans blue leakage through retrograde axonal transport [34]. Likewise, EV-A71-infected tree shrews exhibit reduced Claudin-5 expression as a primary pathological feature [32]. Notably, our study demonstrates that as infection progresses, BBB permeability markedly increases, thereby facilitating viral invasion of the CNS.

The complement system plays a critical role in the pathogenesis of neurological diseases. In herpes simplex virus type 1 (HSV-1) encephalitis, a disease caused by a member of the genus *Simplexvirus*, genetic variants in the *MASP2* gene impair the lectin pathway of complement activation, thereby increasing host susceptibility to HSV-1 infection and accelerating disease progression [35]. Conversely, ZIKV triggers significant upregulation of complement components C1q and C3 in brain, exacerbating neural damage through aberrant complement activation [36]. Furthermore, animal studies have confirmed that elevated C5a in brain aggravates virus-induced brain injury, as observed in models of ZIKV [37] and Middle East Respiratory Syndrome (MERS-CoV; *Betacoronavirus*) infection [38]. Our previous study demonstrated that EV-A71 activates the C5a-C5aR1 axis in astrocytes, which further lead to neuroinflammation [23]. To further investigate the role of the C5a-C5aR1 axis in mediating BBB damage and its underlying mechanisms, we first confirmed the activation of the C5a-C5aR1 axis in human specimens. In C5aR1 receptor knockout mice, we observed a significant reduction in BBB damage induced by EV-A71. There results were in agreement with previous studies showing that targeting C5a-C5aR1 signaling can ameliorate BBB damage and brain injury caused by other microbial infections, including acute sepsis and MERS-CoV [24,38].

C5a is a highly potent chemokine that recruits C5aR1-expressing monocytes and neutrophils to sites of infection [39]. Coxsackievirus A6 (CVA6) is also a member of the genus *Enterovirus* within the family *Picornaviridae*. Our previous work suggests that CVA6 infection is associated with elevated circulating neutrophil levels [40]. Consistently, we observed marked increases in neutrophil counts and NET formation in EV-A71-infected brains. In autopsy samples from children who died of EV-A71-induced encephalitis, there was substantial leukocytes infiltration, with neutrophils prominently localized in perivascular regions and the meninges [10]. Notably, when C5aR1 was knocked out, there was a reduction in the number of neutrophils present in the brain. Supporting these findings, Carvelli et al. reported the involvement of complement C5a and its receptor C5aR1 in disease progression of COVID-19 [41]. In that study, anti-C5aR1 therapeutic monoclonal antibodies blocked the C5a-mediated recruitment and activation of human myeloid cells, and inhibited acute lung injury in human C5aR1 knock-in mice [41].

Neutrophils are the most abundant immune cells in the human body. Their primary function is to undergo cell death as NETs, which capture and eliminate pathogens. However, excessive NET production can trigger uncontrolled inflammatory responses [42]. In recent years, the pathological significance of NETs in human diseases has garnered unprecedented attention. This includes their roles in infectious diseases, sepsis, autoimmune disorders, and various other inflammatory conditions [43–45]. The formation of NETs requires the activation of PAD4 enzyme to induce chromatin decondensation and release [46]. To genetically block NET formation, we utilized PAD4^fl/fl^ and S100A8-Cre mice for the genetic ablation of NETs. As expect, our findings demonstrated that NETs formation promotes EV-A71-induced neuropathogenesis and BBB disruption. NETs function not merely as effectors of local immunity, but rather as facilitators of viral dissemination [47]. The extracellular DNA scaffolds within NETs physically trap viral particles, shielding them from humoral immune clearance and promoting their adhesion to and transmigration across the vascular endothelium. Consequently, specific knockout of PAD4 resulted in a significant reduction in viral load within the bloodstream of EV-A71-infected mice. Furthermore, neutrophils have crucial synergistic roles in thromboinflammation and are increasingly suspected as effector cells contributing to the pathogenesis of neuroinflammatory diseases [48]. Upon severe acute respiratory syndrome coronavirus 2 (SARS-CoV-2; *Betacoronavirus*) infection, neutrophils infiltrate the lungs and form NETs, damaging the lungs and driving an exacerbated immune response [49]. Sivelestat is a small molecule NE inhibitor, often used in the treatment of inflammation in animal disease models and clinical diseases [50]. Current research suggests that sivelestat treatment alleviates SARS-CoV-2-induced lung injury and improves survival in mice [51]. Moreover, in cerebral malaria caused by Plasmodium falciparum infection, sivelestat has been shown to reduce BBB damage and neuroinflammation [52]. Consistently, we showed that early administration of sivelestat significantly mitigated severe illness and brain injury induced by EV-A71. Furthermore, during the early stage of EV-A71 infection, the BBB retains partial integrity, thereby limiting sivelestat penetration into the CNS and resulting in low drug bioavailability at the site of infection. Consequently, future studies will investigate nanoparticle-based delivery systems and combination therapies with other neutrophil elastase inhibitors in humanized mouse models to overcome this pharmacokinetic limitation and better recapitulate clinical pathophysiology [53].

Our data demonstrate that significant BBB leakage was absent within 48 hpi, whereas complement activation (C3/C5a) was evident as early as 6–12 hpi [23]. Consistent with our earlier findings, EV-A71 productively infects astrocytes during this early window, establishing astrocyte infection as the initiating event. This temporal dissociation supports the model in which neuroinflammation precedes and drives BBB disruption, rather than the reverse. Mechanistically, EV-A71-infected astrocytes rapidly produce C5a and CXCL1 [23], recruiting neutrophils that release NETs as the key terminal effectors of BBB damage. This is further supported by our intervention data: while αCXCL1 provided partial protection, blocking NETs was significantly more effective [23]. This hierarchy of therapeutic efficacy indicates that astrocyte-derived inflammation initiates the cascade, but C5a-recruited neutrophils and their NETs serve as the dominant drivers of severe BBB disruption and encephalitis.

Our study has several limitations. First, the small sample size of autopsied tissues from EV-A71 cases may constrain the generalizability of our findings. Second, this study employed neonatal mice to establish an infection model. However, the findings may not be readily generalizable to pediatric patients in clinical settings. Future studies will utilize humanized mouse models to improve the translational relevance and extrapolation of the results. Finally, the in *vivo* assessment of BBB disruption via TJs could be complemented by an in *vitro* BBB model to precisely delineate the direct effects of the virus on endothelial cells; this constitutes a key direction for our future work.

### Conclusion

**Fig 8:**
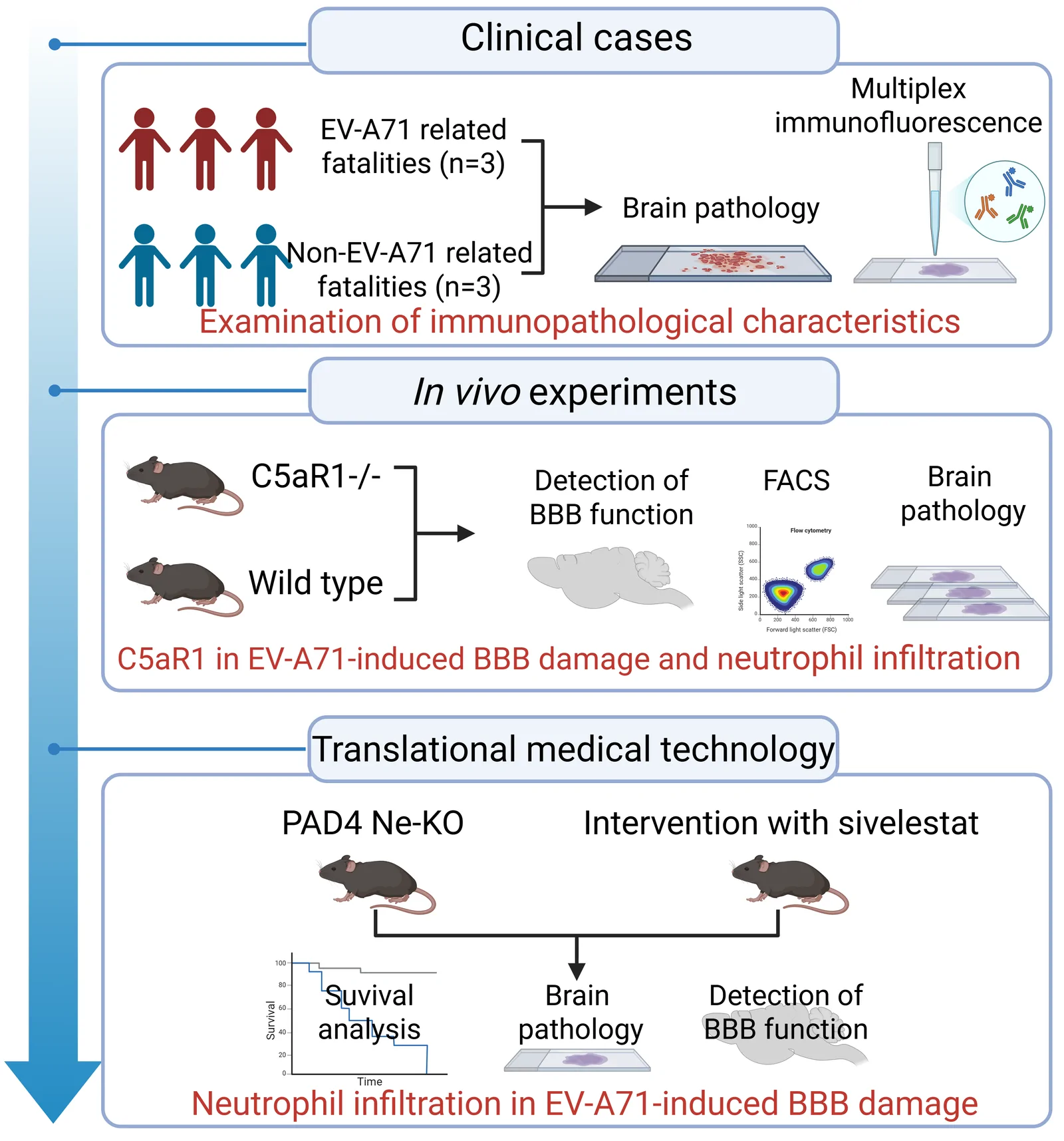
The critical role of the C5a-C5aR1-neutrophil/NETs axis in the pathogenesis of EV-A71 encephalitis. Perivascular neutrophil (CD177+) infiltration and activation of the C5a-C5aR1 axis are observed in the brainstem of EV-A71-associated fatalities. C5aR1 deficiency significantly diminishes both neutrophil infiltration and blood-brain barrier (BBB) damage. Neutrophil-specific PAD4 deletion or pharmacological NET blockade mitigates EV-A71-induced neuroinflammation. EV-A71 infection triggers the intracerebral C5a-C5aR1 axis, promoting neutrophil migration and activation, which in turn exacerbating blood-brain barrier (BBB) damage and neuroinflammation. This illustration was created with BioRender. Jin, **Y.** (2026) https://BioRender.com/hgub59f.

Overall, our study provides evidence that the C5a-C5aR1 axis plays a pivotal role in BBB injury during EV-A71-infected encephalitis. We further delineate the molecular mechanisms by which this axis drives BBB disruption and subsequent neuroinflammation through neutrophil chemotaxis and NET formation. Moreover, our findings highlight sivelestat as a promising therapeutic candidate for EV-A71 encephalitis (Fig 8).

## Materials and methods

### Ethics statement

All experimental protocols involving animal and human specimens were approved by the Life Science Institutional Review Board of Zhengzhou University (ZZUIRBGZR2023–1286) and Kunming Medical University (KMMU2025MEC126). Written informed consent was obtained in advance from the parents/legal guardians of all human participants.

### Cells and virus

Human rhabdomyosarcoma cells were purchased from American Type Culture Collection and maintained in Dulbecco’s modified Eagle’s medium supplemented with 10% fetal bovine serum. The EV-A71 strain with the GenBank accession number OP806304 was described in our previous studies [54,55].

### Animal studies

C57BL/6J mice were obtained from Beijing Vital River Laboratory Animal Technology Co., Ltd. C5aR1 KO mice were purchased from the Jackson Laboratory. C57BL/6NCya-Padi4^em1flox^/Cya (peptidyl arginine deiminase 4, PAD4^fl/fl^) mice and S100A8-Cre (S100A8 is also known as migration inhibitory factor-related protein-8, associated with the activation and migration of neutrophils) mice were purchased from Cyagen Laboratory Animal Science Co., Ltd. These mice were then intercrossed to obtain PAD4^fl/fl^ × S100A8-Cre mice (PAD4 Ne-KO).

Given that infants and young children under five years of age constitute the primary at-risk population for EV-A71 infection, we used 5-day-old neonatal mice to model the susceptible infant population at a corresponding developmental stage. Following established protocols [23], 5-day-old C57BL/6J, C5aR1 KO, PAD4 Ne-KO, and PAD4^fl/fl^ mice were intraperitoneally (i.p.) inoculated with 2.86 × 10^6^ TCID_50_ of EV-A71 to establish infection. Control mice received an equal volume of saline. For the sivelestat intervention, 5-day-old C57BL/6J mice received an intraperitoneal injection of sivelestat (10.0 mg/kg b.w.) at 2 h, 24 h, and 48 h post-EV-A71 infection. Clinical scores and survival of mice were monitored daily until Day 15 p.i.. Clinical scores were evaluated using the following criteria: 0, healthy; 1, lethargy and inactivity; 2, ataxia; 3, loss of weight; 4, hind limb paralysis; 5, dying or dead.

### Human specimens

Brain specimens were obtained from six deceased patients at the School of Forensic Medicine, Kunming Medical University. Among these patients, three were confirmed that they had EV-A71 infection, while the remaining three were uninfected and served as negative controls. The diagnosis of EV-A71-infected fatal cases was conducted in accordance with the Hand-Foot-Mouth Disease Diagnosis and Treatment Guidelines issued by the Chinese Ministry of Health. Patients were assigned to the ‘EV-A71 Infection Group’ based on epidemiological history, clinical manifestations, and laboratory confirmation of EV-A71 infection. Children who died from acute intestinal obstruction or acute enteritis were classified into the ‘Control Group’ by clinicians according to clinical features, laboratory findings, and imaging results. The tissues were fixed in formalin and subsequently embedded in paraffin. In the infection group, there were two males and one female, with a median age of 28 months. Similarly, the control group consisted of two males and one female, with a median age of 22 months (S1 Table).

### Western blotting

Mice brains were homogenized in RIPA lysis buffer containing protease inhibitors. The homogenates were placed on ice for 1 hour, then centrifuged at 12,000 × g for 15 minutes at 4°C. Protein concentrations were determined using a BCA kit. Proteins were separated by 8% or 12% SDS-PAGE and then transferred to PVDF membranes. Membranes were blocked with 5% skim milk, incubated with the primary antibodies, washed three times with TBST, and then incubated with the appropriate secondary antibody. Protein bands were visualized using ECL substrate with the Amersham Imager 600 imaging system (General Electric Co., Ltd. USA). Each blot band was analyzed using Image J software.

### Evans blue extravasation analysis

At day 3, 5, and/or 7 post-infection (p.i.), mice were received 50 µL of Evans blue (20 mg/mL) via tail vein. Two hours later, systemic dye distribution was confirmed by visible blue discoloration of the skin. Mice were then anesthetized with isoflurane, and the thoracic cavity was opened, and transcardiac perfusion was performed with saline until colorless effluent was observed from the right atrial appendage. Brains were then carefully dissected, imaged, and immediately weighed. Each brain was homogenized in 1 mL of formamide, incubated at 60°C for 24 hours, and centrifuged at 10,000 × g for 30 minutes. The absorbance of the supernatant was measured at 620 nm using a spectrophotometer.

### FITC-inulin extravasation analysis

At day 5 p.i., mice received 40 µL of FITC-inulin (10 mg/kg) via tail vein injection. Two hours later, mice were deeply anesthetized with isoflurane and subjected to transcardiac perfusion. Brains were then either imaged under AniView600 Multi-mode Animal Live Imaging System (Guangzhou Biolight Biotechnology Co., Ltd.) or snap-frozen in liquid nitrogen for subsequent immunofluorescence.

### Histology and immunofluorescence (IF)

Brain tissues were collected by trained personnel, fixed in neutral buffered formalin for 24–48 hours, followed by paraffin embedding and sectioning into 4–5 μm slices. Sections were stained with hematoxylin and eosin (H&E), Nissl, or Giemsa for histopathological assessment of brain lesions, or processed for immunostaining with specific antibodies. Brain damage was scored according to established scoring criteria [56]. Images were captured using 3DHISTECH Slide Viewer software.

### RNA extraction, reverse transcription, and RT-qPCR analysis

The brain and blood were homogenized in TRIzol and incubated on ice for 20 minutes. Total RNA was extracted and quantitated using a Nanodrop 2000. cDNA was synthesized with the cDNA Synthesis SuperMix Kit. RT-qPCR was performed using SYBR Green Master Mix on a CFX Opus 96 Real-Time PCR System. Data were analyzed via the 2^-ΔΔCt^ method. RT-qPCR primer sequences are listed in **S2 Table**. All reactions were run in triplicates. Viral load was calculated as described previously [23]. The results are expressed as log_10_ (viral RNA copies) per mg of brain or mL of blood.

### Quantification of myeloperoxidase (MPO)

Brain MPO concentration was quantified using a Myeloperoxidase Assay Kit (Nanjing Jiancheng Bioengineering Institute, China) according to the manufacturer’s instructions.

### Analysis of brain cells by FACS

Flow cytometry was performed as previously described [23]. Brains were mechanically dissociated and filtered through a 70-μm cell strainer to obtain single-cell suspension free of tissue debris. Red blood cells were then lysed, and dead cells were excluded using viability dye. The resulting cell suspension was incubated with fluorochrome-conjugated monoclonal antibodies. Samples were then analyzed on an Agilent NovoCyte flow cytometer, and data were processed using NovoExpress software.

### Antibodies

The following antibodies were used for IF staining, Western blot analysis, FACS: ZO-1, Occludin, and Claudin-5 (Cell Signaling Technologies, Inc., Danvers, Massachusetts, USA); anti-CD177, CD3, CD31, iNOS, CD11b, CD45, IBA1, NeuN, CitH3, HRP-Goat anti-Rabbit IgG, and HRP-Goat anti-Mouse IgG (Servicebio Biotech Co., Ltd., Wuhan, China); anti-C3, C5a/C5a desArg, anti-phosphor-C5aR1 (Ser327), GFAP and β-actin (Abcam Biotechnology, Inc., Cambridge, UK); PerCP/Cyanine5.5 anti-Mouse CD45, Pacific Blue anti-Mouse/Human CD11b, PE anti-Mouse Ly6G (BioLegend, Inc. Japan).

### Statistical analysis

Data were analyzed as means with standard deviations using GraphPad prism 7.0 (GraphPad software Inc., San Diego, CA, USA). Group comparisons used Mann-Whitney U or Student’s *t* test. Survival analysis was conducted using the Kaplan-Meier method, and any disparities in survival rates were evaluated with the log-rank test. For all analysis, difference was considered significant at a *P* < 0.05.

## Supporting information

S1 Fig Quantitative immunofluorescence analysis of immune cells in brains of fatal EV-A71 cases. Positive cell counts in the pons (A) and medulla (B) were quantified using Image J software (n = 3). *P < 0.05; **P < 0.01; ****P < 0.0001 vs. controls.
(DOCX)

S2 Fig Quantitative immunofluorescence analysis of tight junction proteins in brains of fatal EV-A71 cases. Fluorescence intensity of ZO-1 (A), Occludin (B), and Claudin-5 (C) was quantified using Image J software (n = 3). **P < 0.01; ***P < 0.001; ****P < 0.0001 vs. controls.
(DOCX)

S3 Fig Cellular sources of complement in brains of EV-A71-infected mice. Brain sections from EV-A71-infected mice were analyzed by immunofluorescence staining for co-localization of complement proteins with CD45⁺ (leukocytes), GFAP⁺ (astrocytes), IBA1⁺ (microglia), and NeuN⁺ (neurons) (n = 3). Bar = 20 μm. White arrows indicate co-localization.
(DOCX)

S4 Fig Quantitative immunofluorescence analysis of complement proteins in brains of fatal EV-A71 cases. Fluorescence intensity of C3 (A), C5a (B), and phosphor-C5aR1 (Ser327) (C) was quantified using Image J software (n = 3). *P < 0.05; **P < 0.01; ***P < 0.001; ****P < 0.0001 vs. controls.
(DOCX)

S5 Fig Detection of neutrophil extracellular traps in mouse brain tissue. (A, B): CitH3 expression in brain sections was assessed by immunofluorescence using Image J software (n = 3). Bar = 20 μm. Brain dsDNA (C) and NE-DNA (D) levels were detected using commercial kits (n = 5 ~ 6). White arrows indicate positive cells. **P < 0.01; ***P < 0.001; ****P < 0.0001 vs. saline controls.
(DOCX)

S6 Fig Blood viral load in EV-A71-infected mice. (A): Blood viral load in EV-A71-infected C5aR1 KO mice (n = 4). (B): Blood viral load in EV-A71-infected PAD4 Ne-KO mice (n = 4). (C): Blood viral load in sivelestat-treated mice (n = 4). *P < 0.05; ***P < 0.001; ****P < 0.0001 vs. EV-A71-infected control mice.
(DOCX)

S7 Fig NET inhibition reduces brain MPO levels. (A): Brain MPO levels in PAD4 Ne-KO mice (n = 5). (B): Brain MPO levels in sivelestat-treated mice (n = 5). *P < 0.05 vs. EV-A71-infected control mice.
(DOCX)

S1 Table The demographic characteristics of study participants. (DOC)

S2 Table Primers used for RT-qPCR in this study. (DOC)

S1 Data Source data. (XLSX)

S1 File Rawgels. The original blot/gel image data.
(PPTX)
