# Supplementary material for: The C5a-C5aR1 axis facilitates blood-brain barrier disruption induced by EV-A71 infection through neutrophil activation
Source: PLoS Pathog. 2026 Aug 5;22(8):e1014487. doi: 10.1371/journal.ppat.1014487 (PMC13489467; doi:10.1371/journal.ppat.1014487)
Supplement: Supplementary file 1 [file ppat.1014487.s001.docx]

**S1 Fig. Quantitative immunofluorescence analysis of immune cells in brains of fatal EV-A71 cases.** Positive cell counts in the pons (A) and medulla (B) were quantified using Image J software (n = 3). **P* < 0.05; ***P* < 0.01; *****P* < 0.0001 vs. controls.
