# Supplementary material for: The C5a-C5aR1 axis facilitates blood-brain barrier disruption induced by EV-A71 infection through neutrophil activation
Source: PLoS Pathog. 2026 Aug 5;22(8):e1014487. doi: 10.1371/journal.ppat.1014487 (PMC13489467; doi:10.1371/journal.ppat.1014487)
Supplement: Supplementary file 2 [file ppat.1014487.s002.docx]

**S2 Fig. Quantitative immunofluorescence analysis of tight junction proteins in brains of fatal EV-A71 cases.** Fluorescence intensity of ZO-1 (A), Occludin (B), and Claudin-5 (C) was quantified using Image J software (n = 3). ***P* < 0.01;****P* < 0.001; *****P* < 0.0001 vs. controls.
