# Supplementary material for: The C5a-C5aR1 axis facilitates blood-brain barrier disruption induced by EV-A71 infection through neutrophil activation
Source: PLoS Pathog. 2026 Aug 5;22(8):e1014487. doi: 10.1371/journal.ppat.1014487 (PMC13489467; doi:10.1371/journal.ppat.1014487)
Supplement: Supplementary file 3 [file ppat.1014487.s003.docx]

**S3 Fig. Cellular sources of complement in brains of EV-A71-infected mice.** Brain sections from EV-A71-infected mice were analyzed by immunofluorescence staining for for co-localization of complement proteins with CD45⁺ (leukocytes), GFAP⁺ (astrocytes), IBA1⁺ (microglia), and NeuN⁺ (neurons) (n = 3). Bar = 20 μm. White arrows indicate co-localization.
