# Supplementary material for: The C5a-C5aR1 axis facilitates blood-brain barrier disruption induced by EV-A71 infection through neutrophil activation
Source: PLoS Pathog. 2026 Aug 5;22(8):e1014487. doi: 10.1371/journal.ppat.1014487 (PMC13489467; doi:10.1371/journal.ppat.1014487)
Supplement: Supplementary file 4 [file ppat.1014487.s004.docx]

**S4 Fig. Quantitative immunofluorescence analysis of complement proteins in brains of fatal EV-A71 cases.** Fluorescence intensity of C3 (A), C5a (B), and phosphor-C5aR1 (Ser327) (C) was quantified using Image J software (n = 3). **P* < 0.05; ***P* < 0.01; ****P* < 0.001; *****P* < 0.0001 vs. controls.
