# Supplementary material for: The C5a-C5aR1 axis facilitates blood-brain barrier disruption induced by EV-A71 infection through neutrophil activation
Source: PLoS Pathog. 2026 Aug 5;22(8):e1014487. doi: 10.1371/journal.ppat.1014487 (PMC13489467; doi:10.1371/journal.ppat.1014487)
Supplement: Supplementary file 5 [file ppat.1014487.s005.docx]

**S5 Fig. Detection of neutrophil extracellular traps in mouse brain tissue.** (A, B): CitH3 expression in brain sections was assessed by immunofluorescence using Image J software (n = 3). Bar = 20 μm. Brain dsDNA (C) and NE-DNA (D) levels were detected using commercial kits (n = 5~6). White arrows indicate positive cells. ***P* < 0.01; ****P* < 0.001; *****P* < 0.0001 vs. saline controls.
