# Supplementary material for: The C5a-C5aR1 axis facilitates blood-brain barrier disruption induced by EV-A71 infection through neutrophil activation
Source: PLoS Pathog. 2026 Aug 5;22(8):e1014487. doi: 10.1371/journal.ppat.1014487 (PMC13489467; doi:10.1371/journal.ppat.1014487)
Supplement: Supplementary file 6 [file ppat.1014487.s006.docx]

**S6 Fig. Blood viral load in EV-A71-infected mice.** (A): Blood viral load in EV-A71-infected C5aR1 KO mice (n = 4). (B): Blood viral load in EV-A71-infected PAD4 Ne-KO mice (n = 4). (C): Blood viral load in sivelestat-treated mice (n = 4). **P* < 0.05; ****P* < 0.001; *****P* < 0.0001 vs. EV-A71-infected control mice.
