# Supplementary material for: The C5a-C5aR1 axis facilitates blood-brain barrier disruption induced by EV-A71 infection through neutrophil activation
Source: PLoS Pathog. 2026 Aug 5;22(8):e1014487. doi: 10.1371/journal.ppat.1014487 (PMC13489467; doi:10.1371/journal.ppat.1014487)
Supplement: Supplementary file 7 [file ppat.1014487.s007.docx]

**S7 Fig. NET inhibition reduces brain MPO levels.** (A): Brain MPO levels in PAD4 Ne-KO mice (n=5). (B): Brain MPO levels in sivelestat-treated mice (n = 5). **P* < 0.05 vs. EV-A71-infected control mice.
