# Supplementary material for: The C5a-C5aR1 axis facilitates blood-brain barrier disruption induced by EV-A71 infection through neutrophil activation
Source: PLoS Pathog. 2026 Aug 5;22(8):e1014487. doi: 10.1371/journal.ppat.1014487 (PMC13489467; doi:10.1371/journal.ppat.1014487)
Supplement: Supplementary file 8 [file ppat.1014487.s008.doc]

**S1 Table. The demographic characteristics of study participants.**

| **Variables** | EV-A71 (n=3) | Control (n=3) |
| --- | --- | --- |
| Gender (Male, %) | 2 (66.7%) | 2 (66.7%) |
| Age (Median, months) | 28 | 22 |
