# Supplementary material for: The C5a-C5aR1 axis facilitates blood-brain barrier disruption induced by EV-A71 infection through neutrophil activation
Source: PLoS Pathog. 2026 Aug 5;22(8):e1014487. doi: 10.1371/journal.ppat.1014487 (PMC13489467; doi:10.1371/journal.ppat.1014487)
Supplement: Supplementary file 9 [file ppat.1014487.s009.doc]

S2 Table. Primers used for RT-qPCR in this study.

| Gene | Forward | Reverse | Product lengths |
| --- | --- | --- | --- |
| IL-6 | CTGCAAGAGACTTCCATCCAG | AGTGGTATAGACAGGTCTGTTGG | 131 |
| TNF-α | CCTGTAGCCCACGTCGTAG | GGGAGTAGACAAGGTACAACCC | 148 |
| CXCL1 | GCCACACTCAAGAATGGTCG | ACTTGGGGACACCTTTTAGCAT | 94 |
| IL-1β | GAAATGCCACCTTTTGACAGTG | TGGATGCTCTCATCAGGACAG | 116 |
| MCP-1 | TAAAAACCTGGATCGGAACCAAA | GCATTAGCTTCAGATTTACGGGT | 120 |
| VP1 | ATGGTTACGCCAACTGGGAC | CTCTGCATCAAAGCGCATGT | 226 |
| β-actin | GTGCTATGTTGCTCTAGACTTCG | ATGCCACAGGATTCCATACC | 174 |
